# Fat utilization and arterial hypertension in overweight/obese subjects

**DOI:** 10.1186/1479-5876-11-159

**Published:** 2013-07-02

**Authors:** Yvelise Ferro, Carmine Gazzaruso, Adriana Coppola, Stefano Romeo, Valeria Migliaccio, Andrea Giustina, Arturo Pujia, Tiziana Montalcini

**Affiliations:** 1 Clinical Nutrition Unit, Department of Medical and Surgical Science, University Magna Grecia, Viale S. Venuta, 88100 Catanzaro, Italy; 2 Diabetes, Endocrine-metabolic Dis. Cardiovasc. Prevention Unit, Clinical Inst. “Beato Matteo”, Vigevano, Italy; 3 Department of Internal Medicine, I.R.C.C.S. Policlinico San Donato Milanese, Milan, Italy; 4 Department of Molecular and Clinical Medicine, Sahlgrenska Center for Cardiovascolar and Metabolic Research, University of Gothenburg, Gothenburg, Sweden; 5 Department of Biomedical Sciences and Biotechnologies, University of Brescia, Brescia, Italy

**Keywords:** Obesity, Indirect calorimetry, Respiratory Quotient, Hypertension

## Abstract

**Background:**

The Respiratory Quotient is a parameter reflecting the utilization of the nutrients by a subject. It is associated with an high rate of subsequent weight gain and with the atherosclerosis. Subjects tending to burn less fat have an increased Respiratory Quotient. Aim of this study was to investigate on the relationship between the Respiratory Quotient and the cardiovascular risk factors.

**Methods:**

In this cross-sectional study we enrolled 223 individuals of both sexes aged 45–75 ys that were weight stable, receiving a balanced diet, and not affected by debilitating disease or cardiovascular disease. The Respiratory Quotient was measured by Indirect Calorimetry. The measurement of the Blood Pressure was obtained by a mercury sphygmomanometer.

**Results:**

We enrolled 133 female and 90 male. Systolic blood pressure only was positively correlated to the Respiratory Quotient in univariate and multivariate regression analysis (p=0,017). The prevalence of hypertension was significatively different between the quartiles of the Respiratory Quotient, with the highest prevalence in the IV quartile (p=0,024).

**Conclusion:**

High value of the Respiratory Quotient, an index of nutrients utilization, is associated to an high prevalence of Hypertension. It is possible that in the subjects with high Respiratory Quotient and high body mass index, the activation of the renin angiotensin system, in concert to the reduction of the utilization of the endogenous fat stores, could increase the risk of hypertension.

## Introduction

According to the current demographic projections it is expected an increase in the cardiovascular diseases (CVDs) incidence; therefore more sensitive predictors of the risk of the CVDs are needed. In this regard, the Respiratory Quotient (RQ), the ratio between carbon dioxide production and oxygen consumption, reflecting the utilization of the nutrients in a subject [1] may have an important role. In fact, it is well known that after an overnight fast, a subject receiving a balanced diet, that meets the energy requirements for the weight maintenance, burns fat as main substrate; as consequence the value of RQ, usually, results close to 0.85 [1,2]. It has been also shown that subjects tending to burn more glucose, but less fat, have an increased value of RQ [3,4]. Moreover an high RQ is associated with an high rate of subsequent weight gain [2] and with an increased Carotid Intima-Media Thickness (CIMT), a well known predictor of cardiovascular events [5], in overweight/obese individuals of both sexes [6].

At this time, whether RQ is also associated with the cardiovascular risk factors is not fully clarified, therefore aim of this study was to investigate on the relationship between the RQ and the cardiovascular isk factors in individuals of both sexes.

## Method

In our cross-sectional study, we consecutively enrolled 223 individuals among a total of 250 evaluated from January 2011 to September 2012, all undergoing the nutritional screening tests including an Indirect Calorimetry for the RQ and Resting Metabolic Rate (RMR) measurement, at our Clinical Nutrition Unit. The population included both gender, having more than 45 years, with a wide range of body mass index (BMI), and participating in the study on the adherence to Mediterranean Diet and body composition (approved by local ethical committee, projects codes 2011.4; 2013-1/CE ). We enrolled subjects who were weight stable in the four weeks preceding the screening tests, who were following a nutritionally balanced diet meeting energy requirements (i.e.: a solid-food diet that supplied 50% of the calories as carbohydrate, 30% as fat, and 20% as protein), as resulted from the nutritional intake assessment.

According to the medical history, physical examination and blood screening tests, we enrolled apparently healthy individuals. We excluded individuals having clinical evidence of debilitating diseases, (cancer, severe renal failure, sever liver insufficiency, chronic obstructive pulmonary disease), thyroid dysfunction, and cardiovascular disease (myocardial infarction, stroke). We excluded also subjects taking antiobesity medications, psychotropic drugs and chronotropic agents. Furthermore, we excluded individuals following who practiced a regular physical exercise program, who had recently changed the use of tobacco (in the previous four months) and smokers. To avoid the presence of individuals with alterations of the pulmonary ventilation we excluded from the statistical analysis, those having an RQ value over 1.00 or under 0.70 (normal range of RQ: 1.00 to 0.70) [7-9]. The following criteria were used to define the distinct cardiovascular risk factors; diabetes: fasting blood glucose ≥126 mg/dl or antidiabetic treatment; hyperlipidemia: total cholesterol >200 mg/dl and/or triglycerides >200 mg/dl or lipid lowering drugs use; hyperuricemia: serum urate concentration > 7 mg/dl or urate lowering drugs use; hypertension: systolic blood pressure ≥140 mmHg and/or diastolic blood pressure ≥90 mmHg or antihypertensive treatment; overweight: BMI ≥ 25 <30 Kg/m^2^; obesity: body mass index ≥30 kg/m^2^; smoking: current smokers or past smokers (at least before the previous four months) [10,11]. Furthermore, if at least 3 of the NCEP criteria were present [12], the subjects were identified as having the Metabolic Syndrome (MetS).

All tests were performed after a 12 h overnight fasting. Before tests, we gave no particular suggestions on the menu for the meal, but we requested that the dinner before the experiments would have to include types of foods and drinks usually consumed. Indeed they have no caffeinated beverages between their evening meal and the conclusion of the tests on the examination’s morning. Written informed consent was obtained. The investigation conforms to the principles outlined in the Declaration of Helsinki.

### Nutritional intake and anthropometric measurements

The participant’s nutritional intake was calculated using the nutritional software MetaDieta 3.0.1 (Meteda srl, S. Benedetto del Tronto, Italy). Body weight was measured before breakfast with the subjects lightly dressed, subtracting the weight of clothes. Body weight was measured with a calibrated scale and height measured with a wall-mounted stadiometer. BMI was calculated with the following equation: weight (kg) /height (m)^2^. Waist and hip circumferences (WC and HC) were measured with a nonstretchable tape over the unclothed abdomen at the narrowest point between the costal margin and iliac crest and over light clothing at the level of the widest diameter around the buttocks, respectively, as described in the past [13]. Bioelectrical impedance analysis (BIA) (BIA-101; Akernsrl, Florence, Italy) was performed to estimate the Total Body Water (TBW), Fat Mass (FM), Muscle Mass (MM), and total Fat-Free Mass (FFM) [14].

### Blood pressure measurement

The measurement of the systemic BP of both arms was obtained by a mercury sphygmomanometer(systolic blood pressure - SBP and diastolic blood pressure - DBP) as previously described [14,15]. Clinic BP was obtained in the supine patients, after 5 min of quiet rest. A minimum of three BP readings were taken using an appropriate BP cuff size (the inflatable part of the BP cuff covered about 80 percent of the circumference of upper arm).

### RQ and RMR measurement

Fasting RQ and RMR were measured with the participants in their postabsorptive state in a sedentary position. Respiratory gas exchange was measured by Indirect Calorimetry using the open circuit technique between the hours of 7 AM and 8:30 AM after 48-h abstention from exercise. The Indirect Calorimetry instrument (Viasys Healthcare, Hoechberg, Germany) was used for all measurements. The participant rested quietly for 30 min in an isolated room with temperature controlled (21–24°C) environment. The subject was then placed in a ventilated hood for at least 30 min, until steady state was achieved. Criteria for a valid measurement was a minimum of 15 min of steady state, with steady state determined as less than 10% fluctuation in minute ventilation and oxygen consumption and less than 5% fluctuation in RQ. RQ was calculated as CO_2_ production/O_2_ consumption [16].

#### Biochemical evaluation

Venous blood was collected after fasting overnight into vacutainer tubes (Becton & Dickinson) and centrifuged within 4 h. Serum glucose, creatinine, total cholesterol, high density lipoprotein (HDL)-cholesterol, triglycerides, uric acid were measured with Enzymatic colorimetric test. Quality control was assessed daily for all determinations.

#### Statistical analysis

Data are reported as mean ± S.D. The T-test and ANOVA were used to compare the means between groups. The *χ*2-test was used to compare the prevalence among the groups. The univariate analysis was used to determine all the factors correlated to the RQ. In this analysis the following factors were included: age, SBP, DBP, BMI, WC, HC, TBW, FM, MM, FFM, RMR, glucose, total cholesterol, triglycerides, HDL-cholesterol, creatinin, uric acid. The multivariate stepwise regression analysis was used to test for confounding variables. In particular, the variables included in this analisys were all that correlated to the RQ at univariate analisys with a p < 0,1 (model I); furthermore, in a second model (model II) we included also the presence of the Metabolic Syndrome (MetS) *per sè.*

Individuals with incomplete data were excluded from analisys. Significant differences were assumed to be present at *P* < 0.05. All comparisons were performed using the SPSS 17.0 for Windows (Chicago, USA).

## Results

**Figure 1:**
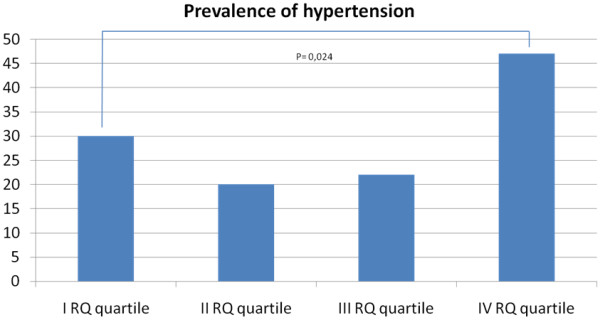
Prevalence of Hypertension among RQ quartiles.

In this study we enrolled 133 female and 90 male with a mean age of 53 ± 12 ys with complete data. There were no differences in RQ value between gender (p= 0,26), therefore all the analyses were performed in the overall population. At univariate analysis we found a positive relation (with a p < 0,1) between RQ and RMR, glucose, triglyceride, SBP and DBP (Table 1). The multivariate regression analysis confirmed that SBP only was correlated to RQ (Table 2, model I). This correlation was confirmed after the presence of the MetS was included in this analysis (Table 2, model II, data not shown). Table 3 shows the characteristics of the population according to RQ quartiles. A significant difference in the prevalence of hypertension was showed between groups, with the highest prevalence in the IV quartile (Figure 1; Table 3). The ANOVA test confirmed that SBP was the only factors significatively different between quartiles (I vs IV ; II vs IV quartile) (Table 3).

**Table 1:** Univariate analysis - factors correlated to RQ

**Variables**	**r**	**p**
RMR	0,138	0,039
glucose	0,135	0,083
trygliceride	0,137	0,076
SBP	0,165	0,031
DBP	0,128	0,095

**Table 2:** Multivariate analysis – factors correlated to RQ

Variables*§	**B**	**SE**	**beta**	**t**	**p**
SBP	0,771	0,038	0,187	2,411	0,017

* the variables included in the model I were: RMR (ns), glucose(ns), triglyceride(ns), SBP, DBP(ns).

§ the variables included in the model II were: RMR (ns), glucose(ns), triglyceride(ns), SBP, DBP(ns), MetS (ns).

**Table 3:** -Characteristics of the population according to RQ quartiles (means and prevalences)

**Variables**	**I RQ quartile (0,78 ± 0,01) (N= 53)**	**II RQ quartile (0,83 ± 0,01) (N=63)**	**III RQ quartile (0,87 ± 0,01) (N=48)**	**IV RQ quartile (0,93 ± 0,02) (N=59)**	**p**
Age (years)	53 ±13	52 ± 12	53 ± 11	53 ± 11	0,922
BMI	33 ± 5	31 ± 5	31 ± 4	32 ± 5	0,053
RMR (kcal)	1509 ± 252	1433 ± 249	1535 ± 251	1571 ± 352	0,051
WC (cm)	106 ± 13	102 ± 11	100 ± 8	106 ± 14	0,064
HC (cm)	109 ± 9	109 ± 10	106 ± 7	111 ± 9	0,160
Glucose (mg/dl)	94 ± 25	95 ± 24	95 ± 21	102 ± 34	0,489
Creatinin (mg/dl)	0,79 ± 0,23	0,76 ± 0,14	0,77 ± 0,2	0,87 ± 0,76	0,595
T Cholesterol (mg/dl)	202 ± 45	205 ± 44	208 ± 44	209 ± 52	0,896
LDL (mg/dl)	126 ± 35	127 ± 40	133 ± 41	129 ± 46	0,911
HDL (mg/dl)	53 ± 17	52 ± 12	46 ± 12	50 ± 16	0,162
Triglyceride (mg/dl)	120 ± 59	130 ± 61	163 ± 127	150 ± 83	0,106
Uric acid (mg/dl)	5,25 ±1,10	5,15 ± 1,60	5,11 ± 1,40	4,95 ± 1,46	0,809
SBP (mmHg)	123 ± 11	123 ± 15	124 ± 13	130 ± 16	0,038
DBP (mmHg)	76 ± 7	74 ± 9	75 ± 9	79 ± 9	0,051
*Prevalences* (%)					
Male	41,5	35	45,8	41	0,705
Hypertension	30	20	22	47	0,024
Diabetes Mellitus	7,5	6,4	5,7	8,9	0,948
Hyperlipidemia	54	54,3	58,3	54,3	0,976
Obesity	68	56	55,3	69	0,263
Metabolic Syndrome	0	2	6,7	5,1	0,665
Medications	4	4	5	5	0,801

## Discussion

The main finding of this study was the direct correlation between RQ and SBP and RMR (Table 1) and the high prevalence of hypertension among individuals with the highest RQ (Table 3, Figure 1). After the adjustments for the confounding factors, the RQ remains correlated to SBP.

In particular, subjects with an RQ higher than 0,90 (IV RQ quartile) had an high prevalence of hypertension (47%) unless their metabolic risk profile was similar to that of the other quartiles (i.e. 40% male, mean age ~ 53 ys and mean BMI ~ 32 Kg/m^2^; tab 3).

This is a novel finding, never investigated to date and very intriguing. It is well known that RQ is associated with a high rate of subsequent weight gain [2,17]. Indeed the obesity rarely exists as an isolated condition, but it is frequently associated with hypertension [18]. Therefore one possible explanation of the link between the high RQ and hypertension may be a high BMI among the subjects with high RQ, [2,19], but in our study the similar BMI among quartiles would reject this explanation. Thus, we infer the presence of other mechanisms like the activation of the Renin Angiotensin System (RAS)[19-27], that results to be associated to the mechanisms of lipid oxidation. Several experiments on mice lacking the Angiotensin II receptor showed, in fact, an increase in the fat utilization and a parallel reduction of the fat mass [28], corroborating the association between hypertension and the index of low fat utilization in our investigation. In addition, a study has shown that in mice with myocardial hypertrophy, the genes associated with the transport and breaking down of the fatty acids are less active than in normal mice [29]. Thus, it is possible to theorize that the failure in the fat utilization may be a predictor of the development of CVDs in individuals with hypertension [29].

It is well accepted that the cardiovascular risk factors are excellent predictors of the CVDs and that their presence may lead to the abnormalities in the structure and function of the cardiovascular system, by a number of complex mechanisms [11,30-34].

We believe that, if confirmed by further investigations, also high RQ may have a role in the clinical practice because it has the potential to identify the effect of the modification in the nutrients utilization on the vascular system. Understanding this concept may give a new view on the mechanisms of the organ damage and their prevention and treatment. Consequently, the RQ measurement may contribute to important changes in the prediction of cardiovascular disease. RQ evaluation probably may identify the individuals needing special therapeutic strategies, like individuals with hypertension. However, because our findings were obtained from cross-sectional data, this hypothetical clinical impact can be emphasized but it is not definitely proved. The RQ measurement offers the potential of a fast analyses using a low cost instrument that is easy to use after a short period of training. However, due to the complexity of the various ways in which different diet may be metabolized, actually there is the need to perform the experiments in the same controlled conditions in all individuals (for example, the same diet).

A limitation of this investigation is the cross-sectional nature, thus this study is not able to determine temporality. Furthermore, probably the serum insulin could contribute to help us to better explain our finding but unfortunately in this study this parameter was lacking. The lack of correlation between the parameters of body composition (WC, HC,TBW, FM, MM, FFM) or the presence of MetS with RQ was expected, on the basis of the similar BMI among quartiles. This finding gives strength to our hypothesis, for which the alteration of some mechanisms of fat utilization is linked to RAS and blood pressure and not to obesity or MetS *per sè.* Of course future studies with a prospective design are needed to clarify the usefulness of the RQ evaluation for the cardiovascular risk stratification and for the prediction of the onset of hypertension.

## Abbreviations

RQ: Respiratory Quotient; CIMT: Carotid intima media thickness; RMR: Resting Metabolic Rate; WC: Waist circumference; BMI: Body mass index; SBP: Systolic blood pressure; DBP: Diastolic blood pressure.

## Competing interests

All authors state that there is no conflict of interest that could be perceived as prejudicing the impartiality of the research reported and all the authors.

## Authors’ contributions

TM and AP were responsible for study design, data analysis, manuscript writer; YF, VM and AC were responsible for integrity of data, data collection and they performed anthropometric measurement and nutritional data collection; SR, CG and AG revised manuscript and approved final version. All authors read and approved the final manuscript.
